# Relationship between HbA1c and Continuous Glucose Monitoring in Chinese Population: A Multicenter Study

**DOI:** 10.1371/journal.pone.0083827

**Published:** 2013-12-23

**Authors:** Jian Zhou, Yifei Mo, Hong Li, Xingwu Ran, Wenying Yang, Qiang Li, Yongde Peng, Yanbing Li, Xin Gao, Xiaojun Luan, Weiqing Wang, Yun Xie, Weiping Jia

**Affiliations:** 1 Department of Endocrinology and Metabolism, Shanghai Jiao Tong University Affiliated Sixth People's Hospital, Shanghai Clinical Center for Diabetes, Shanghai Diabetes Institute, Shanghai Key Laboratory of Diabetes Mellitus, Shanghai Key Clinical Center for Metabolic Disease, Shanghai, China; 2 Department of Endocrinology and Metabolism, Sir Run Run Shaw Hospital, College of Medicine, Zhejiang University, Hangzhou, China; 3 Department of Endocrinology and Metabolism, West China Hospital, Sichuan University, Chengdu, China; 4 Department of Endocrinology and Metabolism, China-Japan Friendship Hospital, Beijing, China; 5 Department of Endocrinology and Metabolism, The Second Affiliated Hospital of Harbin Medical University, Harbin, China; 6 Department of Endocrinology and Metabolism, Shanghai Jiao Tong University Affiliated First People's Hospital, Shanghai, China; 7 Department of Endocrinology and Metabolism, The First Affiliated Hospital of Sun Yat-Sen University, Guangzhou, China; 8 Department of Endocrinology and Metabolism, Fudan University Affiliated Zhongshan Hospital, Shanghai, China; 9 Department of Endocrinology and Metabolism, The First People's Hospital of Foshan, Foshan, China; 10 Shanghai Clinical Center for Endocrine and Metabolic Diseases, Shanghai Institute of Endocrinology and Metabolism, Ruijin Hospital, Shanghai Jiao Tong University School of Medicine, Shanghai, China; 11 Department of Diabetic Neurology, Metabolic Disease Hospital, Tianjin Medical University, Tianjin, China; University of Michigan Medical School, United States of America

## Abstract

**Objective:**

Since there is a paucity of reference data in the literature to indicate the relationship between HbA1c, and 24 h mean blood glucose (MBG) from continuous glucose monitoring (CGM) in Chinese populations, we described the above relationship in adult Chinese subjects with different glucose tolerance status.

**Methods:**

Seven-hundred-and-forty-two individuals without history of diabetes were included to the study at 11 hospitals in urban areas across China from 2007–2009 and data of 673 subjects were included into the final analysis. Oral glucose tolerance test (OGTT) classified the participants as nondiabetic subjects, including those with normal glucose regulation (NGR; *n* = 121) and impaired glucose regulation (IGR; *n* = 209), or newly diagnosed type 2 diabetes (*n* = 343). All participants completed testing for HbA1c levels and wore a CGM system for three consecutive days. The 24 h MBG levels were calculated. Spearman correlations and linear regression analyses were applied to quantify the relationship between glucose markers.

**Results:**

The levels of HbA1c and 24 h MBG significantly increased with presence of glucose intolerance (NGR<IGR<type 2 diabetes; both, *P*<0.001). Analysis of the total population indicated that HbA1c was strongly correlated with 24 h MBG (*r* = 0.735). The correlation was also found to be significant for the subgroup of participants with newly diagnosed type 2 diabetes (*r* = 0.694, *P*<0.001). Linear regression analysis of the total study population yielded the following equation: 24 h MBG _mmol/L_ = 1.198×HbA1c–0.582 (24 h MBG _mg/dL_ = 21.564×HbA1c–10.476) (*R^2^* = 0.670, *P*<0.001). The model fit was not improved by application of exponential or quadratic modeling. When HbA1c was 6.5%, the calculated 24 h MBG was 7.2 (6.4–8.1) mmol/L (130 (115–146) mg/dL); and when HbA1c was 7.0%, the 24 h MBG was 7.8 (6.9–8.7) mmol/L (140 (124–157) mg/dL).

**Conclusions:**

Our study provided the reference data of the relationship between HbA1c and CGM in Chinese subjects.

## Introduction

The prevalence of diabetes in China has increased significantly in the past few years. The 2007 national survey in China reported that the prevalence of diabetes was 9.7% [1]. The recent study by 2010 China Noncommunicable Disease Surveillance Group estimated that the prevalence of Chinese adult diabetes was 11.6%, accounting for 113.9 adults with diabetes [2]. Therefore, diabetes has become a major public health problem in China, with the potential for a major epidemic of diabetes-related complications, including diabetic retinopathy, chronic kidney disease, cardiovascular disease and stroke in China in the near future.

Clinical trials have demonstrated the association between HbA1c and both microvascular and macrovascular complications in type 1 and type 2 diabetes, respectively [3], [4]. Therefore, HbA1c remains the mainstay of monitoring glycemic control during diabetic therapy in China, as well as a feasible approach for diagnosing diabetes in Chinese populations, both from an economic and practical standpoint [5], [6]. HbA1c estimates glucose level over the previous 2–3 months, while the relatively recent development of continuous glucose monitoring (CGM) devices has provided a continuous glycemic profile over a few days and best represents the individual's current glycemic status [7].

CGM provides various glucose information including the glucose patterns, trends and time of changing. The intermittent use of CGM systems was recommended in both adult and pediatric patients with diabetes to detect nocturnal hypoglycemia, dawn phenomenon, postprandial hyperglycemia, and in the management of hypoglycemic unawareness. Recent meta-analysis studies suggested that real-time CGM can be more effective than self monitoring of blood glucose (SMBG) in type 1 diabetes. Also, CGM provided better glycemic control in type 2 diabetic adults when compared with SMBG [8]–[10]. In 2009, our study group established the normal reference values for CGM parameters and recommended a 24 h mean blood glucose (MBG) value<6.6 mmol/L (119 mg/dL) as normal range for the Chinese population [11].

Translating HbA1c into estimated average blood glucose (eAG) has been a major study focus recently. Although the relationship between the mean glucose level and the level of HbA1c has been investigated in several studies, most of the studies either focus on type 1 diabetes or relied on infrequent capillary glucose measurements [12], [13]. For example, the Diabetes Control and Complications Trial (DCCT) described the relationship between HbA1c and eAG based on daily 7-point profiles, and the study was done only in type 1 diabetes [12]. Another multicenter study, the A1c-Derived Average Glucose (ADAG) study assessed a combination of CGM and frequent capillary glucose testing, and HbA1c levels over time to estimate the relationship between the two [14]. However, the withdrawal of one large Asian center in the ADAG study hampered its application in Asian population.

At present, the American Diabetes Association (ADA) has called for laboratories to express HbA1c results as eAG and encouraged clinicians to carry out additional studies to investigate the relationship between HbA1c and eAG in less-studied populations, such as various ethnic groups. Since there remains a paucity of reference data in the previous literature in Chinese populations, we carried out this multicenter study to explore an approach to suggest a relationship between HbA1c level and 24 h MBG by CGM in Chinese subjects with different glucose tolerance status.

## Materials and Methods

### Ethics Statement

This study was independently approved by the local ethics committees of the following hospitals: Shanghai Jiao Tong University Affiliated Sixth People's Hospital, Sir Run Run Shaw Hospital, West China Hospital, China-Japan Friendship Hospital, The Second Affiliated Hospital of Harbin Medical University, Shanghai Jiao Tong University Affiliated First People's Hospital, The First Affiliated Hospital of Sun Yat-Sen University, Fudan University Affiliated Zhongshan Hospital, The First People's Hospital of Foshan, Ruijin Hospital Shanghai Jiao Tong University School of Medicine, and Metabolic Disease Hospital Tianjin Medical University in accordance with the principle of the Helsinki Declaration II. All study participants provided written informed consent upon enrollment and prior to study participation.

### Study Population

A total of 742 study subjects were originally recruited from 11 urban healthcare centers in China between 2007 and 2009. Eligibility criteria for study enrollment included no history of diabetes, hypertension, dyslipidemia, coronary artery diseases or cerebral stroke. Exclusion criteria were as follows: (1) use of medications that may affect glucose metabolism, such as glucocorticoids, thyroid hormones and thiazide diuretics, in the month prior to study enrollment; (2) presence of hepatic dysfunction, defined as >1.5-fold elevated alanine aminotransferase, aspartate aminotransferase, or direct bilirubin; (3) presence of renal dysfunction, defined as serum creatinine >115 µmol/L; (4) women planning for pregnancy during the study period; (5) presence of any conditions or treatments that might interfere with the measurement of HbA1c levels, including but not limited to hemoglobinopathies, anemia (hematocrit <39% in men, and <36% in women), high erythrocyte turnover (evidenced by reticulocytosis), blood loss and/or transfusions, and chronic renal or liver disease, or high-dose vitamin C intake and erythropoietin treatment; or (6) positive results on islet cell antibodies test and/or glutamic acid decarboxylase antibodies test. We excluded 49 subjects, comprising medication users (n = 12), subjects with anemia (n = 27), chronic kidney disease (n = 2) and hepatic dysfunction (n = 8).The remaining 693 subjects were included in the study.

### CGM and Biochemical Measurements Collection

All study participants were fitted with a CGM device (MiniMed; Medtronics, Northridge, CA, USA) for continuous measurements of subcutaneous interstitial glucose over three consecutive days (day 0, insertion of the CGM sensor; day 3, removal of sensor). The CGM system was calibrated daily by the participant by entering a minimum of four capillary blood glucose readings that had been obtained with a blood glucose meter (SureStep; LifeScan, Milpitas, CA, USA). A total of 288 blood glucose values were obtained for each 24 h period (with 5 min intervals), which were used to calculate the daily 24 h MBG level; and the participant's overall 24 h MBG was based on the values for days 1 and 2.

The following criteria for optimal accuracy were adhered to: a mean absolute difference of ≤28% when the daily range of meter values was ≥5.6 mmol/L (100 mg/dL) and a mean absolute difference of ≤8% when the daily range of meter values was <5.6 mmol/L(100 mg/dL). The detailed protocols for CGM have been described previously [11].

On day 4, fasting plasma glucose (FPG) and HbA1c levels were measured after at least a 10-hour overnight fast among all study participants, and a 75-g oral glucose tolerance test (OGTT) was then conducted among participants. HbA1c was measured by high-performance liquid chromatography on the Variant II HbA1c analyzer (Bio-Rad Laboratories, Hercules, CA, USA); the inter- and intra-assay coefficients of variation were <0.4% and <0.6%, respectively [5]. During the standard 75-g OGTT, plasma glucose was measured at 30 min, 1 hour, 2 hours and 3 hours after administration. Blood specimens for the glucose test were collected using vacuum blood–collection tubes containing anticoagulant sodium fluoride and were centrifuged on site within 2 hours of collection. Plasma glucose was measured locally using glucose oxidase within 24 hours.

### Statistical methods

OGTT classified the participants as nondiabetic subjects, including those with normal glucose regulation (NGR) and impaired glucose regulation (IGR), or newly diagnosed type 2 diabetes according to the 2007 diagnostic criteria [15]. The 24 h MBG was analyzed using CGMS Solutions software (version 3; Medtronics), and all other statistical analyses were carried out with the SPSS software suite (version 13.0; SPSS Inc., Chicago, IL, USA). Normally distributed data are presented as mean ± SD, and skewed variables are presented as median (interquartile range: 25th to 75th percentile). Data of clinical characteristics that followed a normal distribution pattern were compared among subgroups of glucose tolerance status by using the one-way analysis of variance with post-hoc LSD test, while those with non-normal distribution were compared using the Kruskal-Wallis test. The significance of inter-group differences of categorical variables was evaluated by Chi-squared test. The correlations between HbA1c, 24 h MBG and plasma glucose levels during OGTT were evaluated using Spearman's correlation coefficient analysis. Multiple linear regression analyses were performed to assess the independent effects of each time-point glucose levels during OGTT on HbA1c and 24 h MBG, respectively. Linear regression modeling was performed to estimate the relationship between HbA1c and 24 h MBG in total subjects; and in subgroups with different gender or different glucose tolerance state. The exponential or quadratic regression models were also performed to compare the fitness of the model with the linear regression model. Prediction intervals were calculated to represent the range of predicted 24 h MBG at given HbA1c levels. Statistical significance was indicated by a two-tailed *P* value of <0.05.

## Results

### Characteristics of study subjects

Twenty participants were excluded due to signal interruption of the CGM system or not meeting the system's accuracy requirements. The data from the remaining 673 enrollees were incorporated into the statistical analysis, including 345 men and 328 women. The mean age was 52±13 years and body mass index (BMI) was 24.85±3.43 kg/m^2^. The total group mean±SD was 6.8±1.5% for HbA1c, and 7.5±2.2 mmol/L (135±40 mg/dL) for 24 h MBG.

All enrollees were classified as normal glucose regulation (NGR; *n* = 121), impaired glucose regulation (IGR; *n* = 209), or newly diagnosed type 2 diabetes (*n* = 343) according to OGTT results. The sex ratios were not significantly different (χ^2^ = 4.309, *P* = 0.116) but the age distribution was significantly different among the subgroups (*F* = 32.089, *P*<0.01). As expected, the levels of HbA1c, FPG, 2-hour postload plasma glucose and 24 h MBG significantly increased in conjunction with the presence of glucose intolerance (NGR<IGR<type 2 diabetes, all *P*<0.001). Compared with NGR category, participants with newly diagnosed diabetes had higher blood pressure, lipid levels and ALT level. Table 1 presents the population characteristics in subgroups with different glucose tolerance status.

**Table 1:** Clinical characteristics at baseline of study participants.

Characteristics	Total	NGR	IGR	NDM	*P* value
	*n* = 673	*n* = 121	*n* = 209	*n* = 343	
Age, y	52 (13)	45 (14)	56 (12)*	53 (12)* ^†^	<0.001
Gender, male/female	345/328	63/58	95/114	187/156	0.116
Body mass index, kg/m^2^	24.8 (3.4)	22.3 (2.0)	24.6 (2.9)*	25.8 (3.6)* ^†^	<0.001
Systolic Blood Pressure, mmHg	120.0 (112.0–135.0)	115.0 (110.0–125.0)	120.0 (118.0–135.0)^‡^	127.0 (120.0–140.0)^‡^	<0.001
Diastolic Blood Pressure, mmHg	80.0 (70.0–82.0)	80.0 (70.0–80.0)	80.0 (70.0–80.0)	80.0 (70.0–85.0)^‡^	0.003
24 h MBG, mmol/L	7.5 (2.2)	5.7 (0.7)	6.6 (0.8)*	8.7 (2.4)* ^†^	<0.001
HbA1c, %	6.8 (1.5)	5.5 (0.4)	6.1 (0.6)*	7.7 (1.5)* ^†^	<0.001
FPG, mmol/L	6.8 (2.2)	4.8 (0.4)	5.9 (0.6)*	8.1 (2.3)* ^†^	<0.001
2-hour postload glucose, mmol/L	11.5 (5.3)	5.3 (1.2)	8.6 (1.5)*	15.9 (4.2)* ^†^	<0.001
Total cholesterol, mmol/L	5.0 (1.1)	4.6 (0.8)	5.1 (1.1)*	5.1 (1.1)*	<0.001
Triglycerides, mmol/L	1.5 (1.1–2.1)	0.9 (0.7–1.4)	1.5 (1.0–2.0)^‡^	1.7 (1.3–2.6)^‡§^	<0.001
HDL-C, mmol/L	1.2 (1.1–1.5)	1.4 (1.2–1.8)	1.3 (1.1–1.5)^‡^	1.2 (1.0–1.4)^‡§^	<0.001
LDL-C, mmol/L	3.0 (0.9)	2.8 (0.9)	3.0 (0.8)	3.1 (1.0)*	0.004
ALT, U/l	24.0 (16.0–36.0)	17.0 (12.0–27.0)	22.0 (15.0–33.0)^‡^	26.0 (18.0–40.0)^‡§^	<0.001
AST, U/l	23.0 (19.0–29.0)	22.0 (18.0–28.0)	23.0 (19.0–28.0)	23.0 (19.0–30.0)	0.583
BUN, mmol/L	5.3 (1.3)	5.2 (1.3)	5.4 (1.3)	5.3 (1.3)	0.228
Plasma creatinine, μmol/l	69.3 (16.6)	69.5 (15.6)	69.9 (17.5)	68.9 (16.2)	0.769
Uric acid, μmol/l	318.1 (84.9)	300.3 (69.7)	331.7 (93.4)*	313.8 (81.6)	0.008

Data are mean (SD), median (25^th^ to 75^th^ percentile) or n.

P<0.05 vs NGR group; ^†^P<0.05 vs IGR group; ^‡^P<0.01 vs NGR group; ^§^P<0.01 vs IGR group

Abbreviations: NGR, normal glucose regulation; IGR, impaired glucose regulation; NDM, newly diagnosed diabetes; FPG, fasting plasma glucose; HDL-C, high density lipoprotein cholesterol; LDL-C, low density lipoprotein cholesterol; ALT, alanine aminotransferase; AST, aspartate aminotransferase; BUN, blood urea nitrogen.

### Correlations among HbA1c or 24 h MBG and FPG, and 2-h postload plasma glucose

In Table 2, Spearman correlation coefficients among HbA1c or 24 h MBG and FPG, 30 min postload plasma glucose, 1-hour postload plasma glucose, 2-hour postload plasma glucose and 3-hour postload plasma glucose are presented. Next, multiple linear regression analyses were performed to assess the independent effects of glucose levels during OGTT on HbA1c and on 24 h MBG. Both FPG and 2-hour postload plasma glucose remained significant in stepwise regression analysis (multiple *R* ^2^ = 0.748 for the model of HbA1c and multiple *R^2^* = 0.730 for the model of 24 h MBG) (Table 3).

**Table 2:** Spearman correlation coefficients among HbA1c and glucose, 24(*n* = 673).

	Fasting plasma glucose	30 min postload plasma glucose	1-hour postload plasma glucose	2-hour postload plasma glucose	3-hour postload plasma glucose
HbA1c	0.781	0.716	0.746	0.774	0.610
24 h MBG	0.785	0.683	0.694	0.748	0.622

all, *P*<0.001.

**Table 3:** Multiple stepwise regression analysis with HbA1c or 24(*n* = 673).

Dependent variable	Explanatory variable	Standardized Regression Coefficient	*t*	*P* value	Adjusted *R* ^2^ of the Model
HbA1c	FPG	0.551	14.602	<0.001	0.748
	2-h postload plasma glucose	0.357	9.455	<0.001	
24 h MBG	FPG	0.647	16.550	<0.001	0.730
	2-h postload plasma glucose	0.242	6.199	<0.001	

Independent factors included fasting plasma glucose, 30 min postload plasma glucose, 1-hour postload plasma glucose, 2-h postload plasma glucose and 3-hour postload plasma glucose.

### Correlations between HbA1c and 24 h MBG

**Figure 1:**
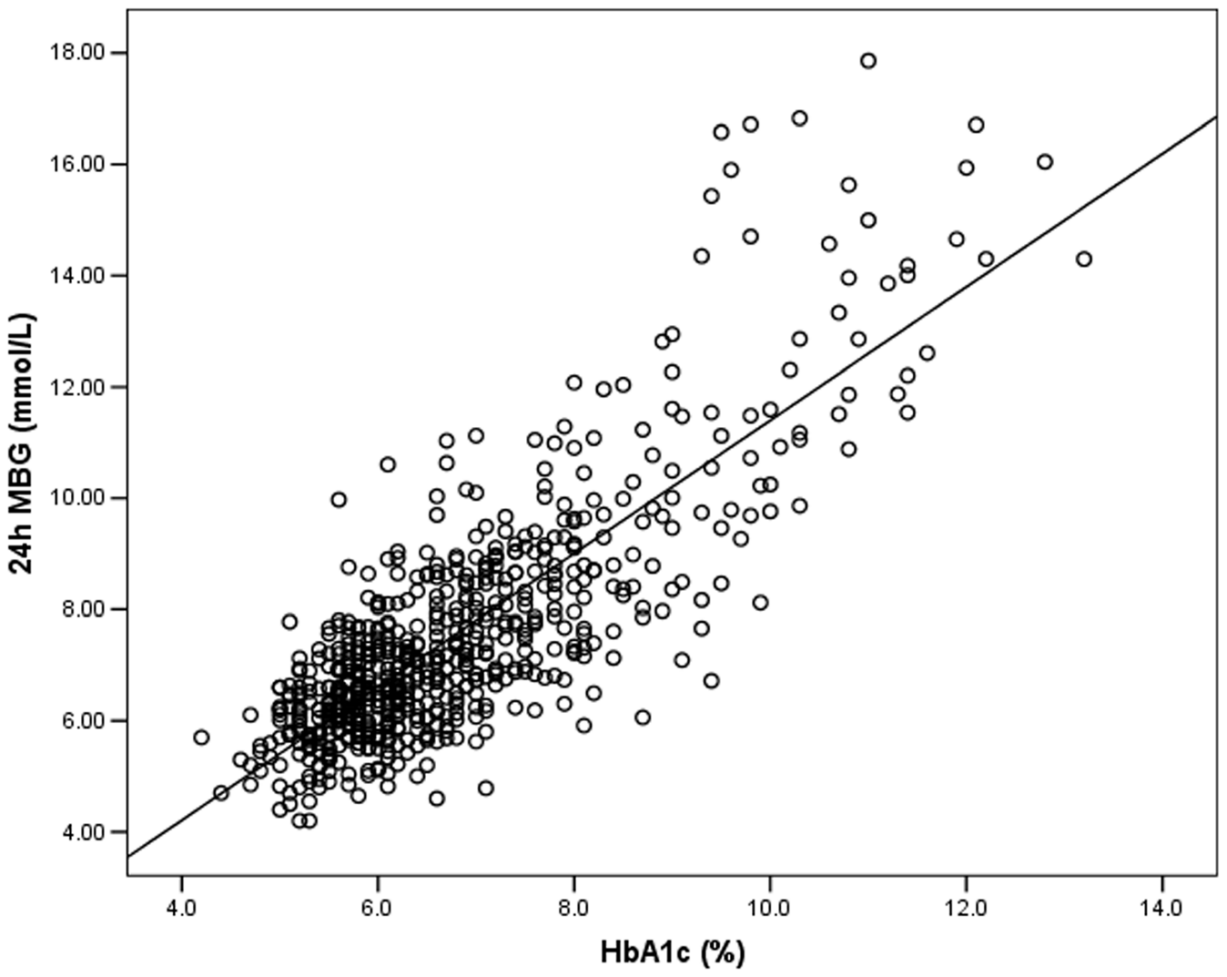
Correlation analysis of 24(*n* = 673). A significant positive correlation exists between the 24(*r* = 0.735, *P*<0.001).

24 h MBG was positively correlated with HbA1c (*r* = 0.735, *P*<0.001) in all subjects (Figure 1). When the population was stratified by sex, the correlation was significant for both (*r* = 0.735 for males and *r* = 0.737 for females, both *P*<0.001). The correlation was also found to be significant for the subgroup of participants with newly diagnosed type 2 diabetes (*r* = 0.694, *P*<0.001). Linear regression analysis of the total study population yielded the following equation: 24 h MBG _mmol/L_ = 1.198×HbA1c –0.582 (*R^2^* = 0.670, *P*<0.001) (24 h MBG _mg/dL_ = 21.564×HbA1c–10.476) (Figure 1). The model fit was not improved by application of exponential or quadratic modeling (data not shown). As estimated from the equation, the mean increase of MBG per 1% increase in HbA1c was 1.2 mmol/L (22 mg/dL). Table 4 shows the translation of HbA1c level to 24 h MBG level based on the linear regression modeling, with 95% prediction limits. Analysis of the subgroup of participants with newly diagnosed type 2 diabetes indicated that the relationship between HbA1c and 24 h MBG was similar to that seen with the total group: 24 h MBG _mmol/L_ = 1.202×HbA1c–0.488 (*R^2^* = 0.601, *P*<0.001) (24 h MBG _mg/dL_ = 21.636×HbA1c–8.784). In this subgroup model, the calculated 24 h MBG was 7.3 (5.8–8.8) mmol/L (131 (104–158) mg/dL) and 7.9 (6.4–9.5) mmol/L (142 (115–171) mg/dL) when HbA1c was 6.5% and 7.0%, respectively.

**Table 4:** The 24(95% CI, *n* = 673).

HbA1c, %	24 h MBG, mg/dL	24 h MBG, mmol/L
5.0	97 (85–112)	5.4 (4.7–6.2)
6.0	119 (104–133)	6.6 (5.8–7.4)
6.5	130 (115–146)	7.2 (6.4–8.1)
7.0	140 (124–157)	7.8 (6.9–8.7)
8.0	162 (146–180)	9.0 (8.1–10.0)
9.0	184 (166–202)	10.2 (9.2–11.2)
10.0	205 (185–225)	11.4 (10.3–12.5)
11.0	225 (207–247)	12.5 (11.5–13.7)
12.0	248 (227–270)	13.8 (12.6–15.0)

Data are presented as mean (95% CI).

Linear regression 24 h MBG _mmol/L_ = 1.198×HbA1c–0.582.

Linear regression 24 h MBG _mg/dL_ = 21.564×HbA1c–10.476.

## Discussion

HbA1c has been proposed by the ADA as an optional assay for diagnosing diabetes and also for detecting individuals at increased risk of the disease [16]. Translation of HbA1c level to eAG level is a well-recognized and widely practiced technique [12], [13], [17]. The ADA has called for laboratories to express HbA1c results as eAG since eAG is easier for patients to understand and will lead to improved management of diabetes in clinical practice. A few studies have examined the relationship and various equations have been attained. In the DCCT study [12], retrospective analysis of data derived from SMBG measurements identified a linear correlation between HbA1c and eAG concentrations. However, the DCCT study was not originally designed to determine eAG, and the correlation was based on only fingerstick glucose measurements. Another study, the ADAG study [14], defined a mathematical equation between HbA1c and the eAG level (eAG_ mg/dL_ =  28.7×HbA1c–46.7), which has been widely used in the clinical practice and the equation was recommended by the ADA's calculation of the estimate average glucose (eAG). In ADAG study, participants underwent CGM for 48 h at baseline and monthly for the duration of the study, as well as the SMBG measurement 7 times per day for at least 3 days per week. Over the course of the 12-week study, approximately 2700 glucose measurements were performed on each participant. Unfortunately, the withdrawal from the ADAG study of one large center serving Asian populations precluded extension of its findings to patients of Asian ethnic groups, such as the Chinese. Therefore, the current cross-sectional study based on Chinese nationals affords the opportunity to establish a set of reference values reflecting the interrelationship between HbA1c and 24 h MBG, which may facilitate future prospective follow-up studies.

The mean increase of 24 h MBG per 1% increase in HbA1c found in our present study (1.2 mmol/L, 22 mg/dL) was lower than that found in either the DCCT study (1.98 mmol/L, 36 mg/dL) [12] or the ADAG study (1.59 mmol/L, 29 mg/dL) [14]. The difference may be attributed to several distinctive characteristics of the three study designs. First, the DCCT study only included subjects with type 1 diabetes, while the ADAG study population was composed of individuals with NGR or both type 1 and type 2 diabetic patients. In contrast, our study population was composed of nondiabetic individuals with NGR or IGR, as well as newly diagnosed diabetes, but only those with type 2 diabetes. Second, the Eastern countries have a different traditional diet and lifestyle when compared to the Western countries, although the former are rapidly adopting those of the later. Intriguingly, a large number of the Chinese subjects in our study who were newly diagnosed with diabetes showed isolated post-challenge hyperglycemia, instead of impaired fasting glucose, which is generally attributed to a carbohydrate-rich diet. Lastly, it is known that racial disparities exist among HbA1c values [18]. There is evidence of wide fluctuations in HbA1c between individuals that are unrelated to glycemic status, suggesting the existence of high and low glycators. High glycators have consistently higher HbA1c than expected for their MBG, whereas low glycators have lower HbA1c than their MBG would suggest [19], [20]. For instance, a recent epidemiologic study found that, when matched for FPG, African Americans had higher HbA1c than Caucasians, suggesting that their glycemic burden may be higher [21]. The possible explanations for this between-individual variability in hemoglobin glycation rate may attribute to the differences in erythrocyte survival and some yet unknown genetic elements [22], [23].

Another interesting finding in the present study is that, significantly higher LDL and systolic blood pressure levels were found among the newly diagnosed diabetes group, as compared to NGR and IGR groups. Hypertension is a common comorbidity of diabetes. In type 1 diabetes, hypertension could be the result of underlying nephropathy, while in type 2 diabetes it often coexists with other risk factors of cardiovascular disease (CVD). Also, type 2 diabetic patients sometimes have an increased prevalence of lipid abnormalities. Several clinical trials have demonstrated significant effects of statin therapy on primary and secondary prevention of CVD events in diabetic subgroup [24], [25]. Indeed, anti-hypertensive treatment, lipid-lowering therapy, and lifestyle modification are still regarded as the primary strategies for reducing the burden of macrovascular complications in diabetes [26].

Nonetheless, several features of the current study's design may have impacted the findings and should be taken into consideration when interpreting our results. The cross-sectional study design, itself, precludes our ability to make any direct conclusions about the relationship. The fact that we did not include patients with type 1 diabetes also limits the generalized ability of our findings to all diabetes. Further studies validating the findings from the present study would be required before any implementation of our results could be considered.

## Conclusions

In conclusion, Chinese adults show a strong correlation of 24 h MBG with HbA1c levels (24 h MBG _mmol/L_ = 1.198×HbA1c–0.582; 24 h MBG _mg/dL_ = 21.564×HbA1c– 10.476). Analysis of nondiabetes and type 2 diabetes indicated that when HbA1c is 6.5% then the calculated 24 h MBG is 7.2 mmol/L (130 mg/dL). Likewise, when HbA1c is 7.0% then the 24 h MBG is 7.8 mmol/L (140 mg/dL). These results may prove useful in future clinical and research applications focused on Asian population, especially Chinese patient cohorts.

## Supporting Information

Text S1 **List of participating investigators.**
(DOC)
