# Supplementary material for: Relationship between HbA1c and Continuous Glucose Monitoring in Chinese Population: A Multicenter Study
Source: PLoS One. 2013 Dec 23;8(12):e83827. doi: 10.1371/journal.pone.0083827 (PMC3871656; doi:10.1371/journal.pone.0083827)
Supplement: Text S1 — List of participating investigators. (DOC) [file pone.0083827.s001.doc]

**Appendix**

**List of participating investigators**

1, Department of Endocrinology and Metabolism, Shanghai Jiao Tong University Affiliated Sixth People’s Hospital, Shanghai Diabetes Institute, Shanghai Clinical Center for Diabetes, Shanghai, China (Weiping Jia, Jian Zhou, Kunsan Xiang, Yuqian Bao, Xiaojing Ma, Wei Lu, Cheng Hu, Huijuan Lu, Yifei Mo)

2, Department of Endocrinology and Metabolism, Sir Run Run Shaw Hospital, College of Medicine, Zhejiang University Hangzhou, China (Hong Li, Fenping Zheng)

3, Department of Endocrinology and Metabolism, West China Hospital, Sichuan University, Chengdu, China (Xingwu Ran, Liping He)

4, Department of Endocrinology and Metabolism, China-Japan Friendship Hospital, Beijing, China (Wenying Yang, Jinping Zhang, Na Wang)

5, Department of Endocrinology and Metabolism, The Second Affiliated Hospital of Harbin Medical University, Harbin China (Qiang Li, Lili Chen)

6, Department of Endocrinology and Metabolism, Shanghai Jiao Tong University Affiliated First People’s Hospital, Shanghai, China (Yongde Peng, Yufan Wang)

7, Department of Endocrinology and Metabolism, The First Affiliated Hospital of Sun Yat-Sen University, Guangzhou, China (Yanbing Li, Juan Liu)

8, Department of Endocrinology and Metabolism, Fudan University Affiliated Zhongshan Hospital, Shanghai, China (Xin Gao, Zhiqiang Lu, Ran You)

9, Department of Endocrinology and Metabolism, The First People’s Hospital of Foshan, Foshan, China (Xiaojun Luan)

10, Shanghai Clinical Center for Endocrine and Metabolic Diseases, Shanghai Institute of Endocrinology and Metabolism, Ruijin Hospital, Shanghai Jiao Tong University School of Medicine, Shanghai, China (Weiqing Wang, Shouyue Sun)

11, Department of Diabetic Neurology, Metabolic Disease Hospital, Tianjin Medical University, Tianjin, China (Yun Xie)
